# Cardiorespiratory arrest after iso-osmolar iodinated contrast injection: A case report of contrast-induced encephalopathy following contrast-enhanced computed-tomography

**DOI:** 10.1097/MD.0000000000024035

**Published:** 2021-01-15

**Authors:** Li-Ding Yao, Xiu-Liang Zhu, Run-Lin Yang, Min-Ming Zhang

**Affiliations:** a Department of Radiology, The Second Affiliated Hospital, Zhejiang University School of Medicine, Hangzhou, China; b School of Medicine, University of Melbourne, Melbourne, Australia.

**Keywords:** cardiorespiratory arrest, contrast-induced encephalopathy, computed-tomography

## Abstract

**Rationale:**

Contrast-induced encephalopathy (CIE) is a rare complication caused by administration of intravascular contrast media and characterized by acute reversible neurological disturbance. Most of the CIE cases are reported after arterial administration of contrast media such as during cerebral or coronary angiographies, yet only a few articles have reported CIE secondary to intravenous contrast. A case of CIE secondary to intravenous contrast administration is reported here.

**Patient concerns:**

A 68-year-old man was admitted to our hospital for contrast-enhanced chest computed-tomography (CT) examination due to suspected pulmonary nodules. After CT examination, the patient lost consciousness and experienced a cardiorespiratory arrest. An emergency plain brain CT was done immediately which showed abnormal cortical contrast enhancement and cerebral sulci hyperdensity.

**Diagnoses:**

After excluding other differential diagnoses such as electrolytes imbalance, hypo/hyperglycemia, cardiogenic pathologies and other neurological emergencies such as cerebral hemorrhage, cerebral infarction, the final diagnosis of CIE was made.

**Interventions:**

The patient was admitted to the intensive care unit for further management. A series of supportive treatments were arranged.

**Outcomes:**

Follow-up visits at the outpatient clinic showed no lasting neurological deficits.

**Lessons:**

CIE should be considered as 1 of the differential diagnoses for a patient with acute neurologic symptoms after iodinate contrast administration. Neuroradiological imaging examinations are essential to rule out other etiologies such as acute cerebral infarction or intracranial hemorrhage.

## 1 Introduction

Contrast-induced encephalopathy (CIE) is a rare complication caused by administration of intravascular contrast media and characterized by acute reversible neurological disturbance.^[1]^ Although it is most commonly seen after cerebral angiography, it has also been reported after contrast-enhanced computed-tomography (CT), cardiac and peripheral angiography.^[2]^ CIE can cause a variety of neurological symptoms ranging from confusion and headache to more serious manifestations such as seizure, transient cortical blindness and focal neurological deficits. Transient cortical blindness is the most frequently seen clinical presentation of CIE.^[1,3]^ The prognosis of CIE can be extremely favorable.^[4]^

Here we report a case of CIE that took a far more devastating course, resulting in a sudden loss of consciousness and cardiorespiratory arrest after chest contrast-enhanced CT.

## 2 Ethical review

### 2.1 Ethical approval

This article does not contain any studies with human or animals performed by any of the authors.

### 2.2 Informed consent

Informed consent was obtained from the patient for publication of this case report and accompanying images.

### 2.3 Case description

A 68-year-old man was admitted to our hospital for contrast-enhanced chest CT examination due to suspected pulmonary nodules. His past medical history included rheumatoid arthritis for more than ten years, for which he received methotrexate treatment. The patient had no history of allergy and had a normal baseline serum creatinine level (70 μmol/L, normal range: 40-106 μmol/L).

**Figure 1:**
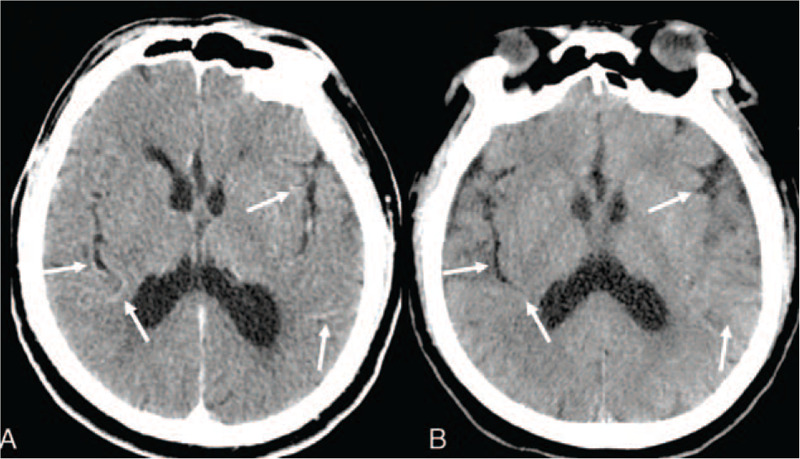
Plain brain CT acquired immediately and 2 days after admission A, Plain brain CT images showed abnormal cortical contrast enhancement and cerebral sulci hyperdensity. B, Plain brain CT images acquired 2 days after admission showed no abnormalities.

During the CT examination, a total of 90 ml (1.2 ml/kg, 75 kg) of iso-osmolar iodinated contrast was given intravenously. By the completion of this procedure, the patient was found to have dysarthria but was conscious. Seconds later, the patient lost consciousness and experienced cardiorespiratory arrest. He received immediate cardiopulmonary resuscitation and tracheal intubation. An emergency plain brain CT was done which showed abnormal cortical contrast enhancement and cerebral sulci hyperdensity (Fig. 1A). Laboratory tests showed normal electrolytes and blood glucose levels. Emergency electrocardiogram showed rapid atrial fibrillation and ST segment elevation, suggesting the possibility of inferior myocardial infarction. Initially, the cardiologists thought the cardiac arrest was due to the myocardial infarction. An emergency coronary angiography was performed afterwards but revealed no abnormalities except for left anterior descending branch myocardial bridge. The patient was then admitted to the intensive care unit for further management. A non-contrast CT was done again on the second day after admission, showing that the high-density lesions had disappeared (Fig. 1B). Lumbar cerebrospinal fluid analysis on day 3 yielded normal findings regarding cell counts and protein content. In particular, neither xanthochromia nor red blood cells were found in cerebrospinal fluid sample, suggesting the exclusion of hemorrhage.

The patient was closely monitored for blood pressure, heart rate and consciousness level in the intensive care unit ward and no deterioration was observed. Notably, after a series of supportive treatments, the patient's condition gradually improved and slowly regained consciousness. On day 10, the patient fully regained consciousness with a Glasgow Coma Scale of E4VTM6 and tracheal intubation was removed.

**Figure 2:**
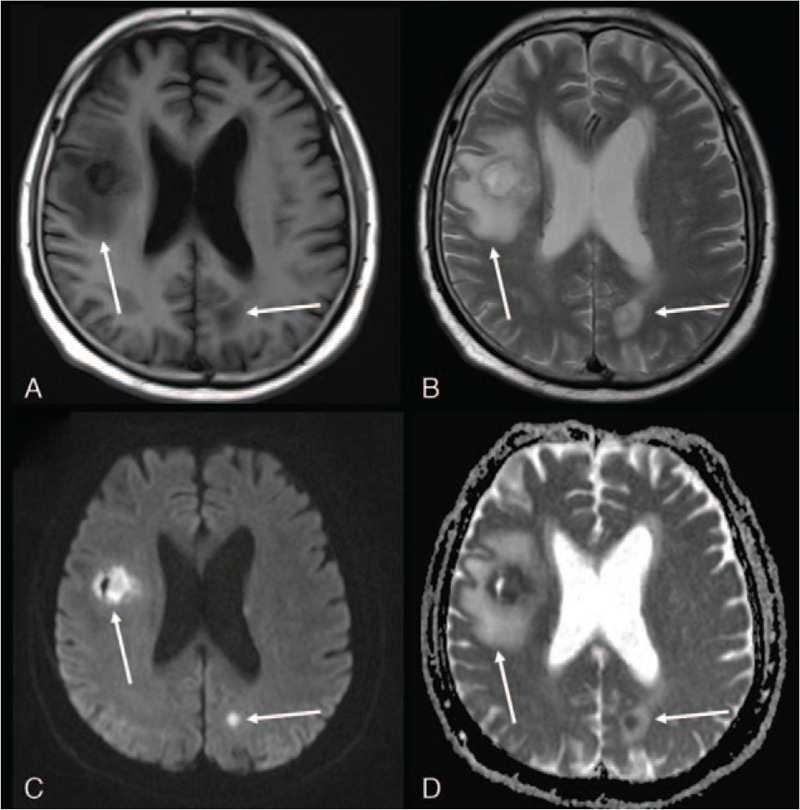
Magnetic resonance imaging acquired on day 17 showed cystic swelling which manifested as centrally hypointense signals on T1 weighted images, hyperintense signals on T2 weighted images with iso-to-low signal border in right frontal lobe and left parietal lobe. The lesions were hyperintense on diffusion weighted images (DWI) and hypointense on apparent diffusion coefficient (ADC) maps indicating restricted diffusion and possible brain abscess. A, Axial T1-weighted image. B, Axial T2-weighted image. C, Diffusion-weighted image (b = 800). D, Apparent diffusion coefficient map.

However, in the next few days, the patient developed fever with elevated neutrophil levels. On day 17, magnetic resonance imaging (MRI) of the brain showed cystic swelling which manifested as centrally hypointense signals on T1 weighted images, hyperintense signals on T2 weighted images with iso-to-low signal border in right frontal lobe and left parietal lobe. The lesions were hyperintense on diffusion weighted images and hypointense on apparent diffusion coefficient maps indicating restricted diffusion and possible brain abscess (Fig. 2). Hence the patient underwent lumbar puncture yet revealed no signs of intracranial infection. The edema around the lesions progressed in the subsequent brain MRI examinations. The patient then developed headache.

**Figure 3:**
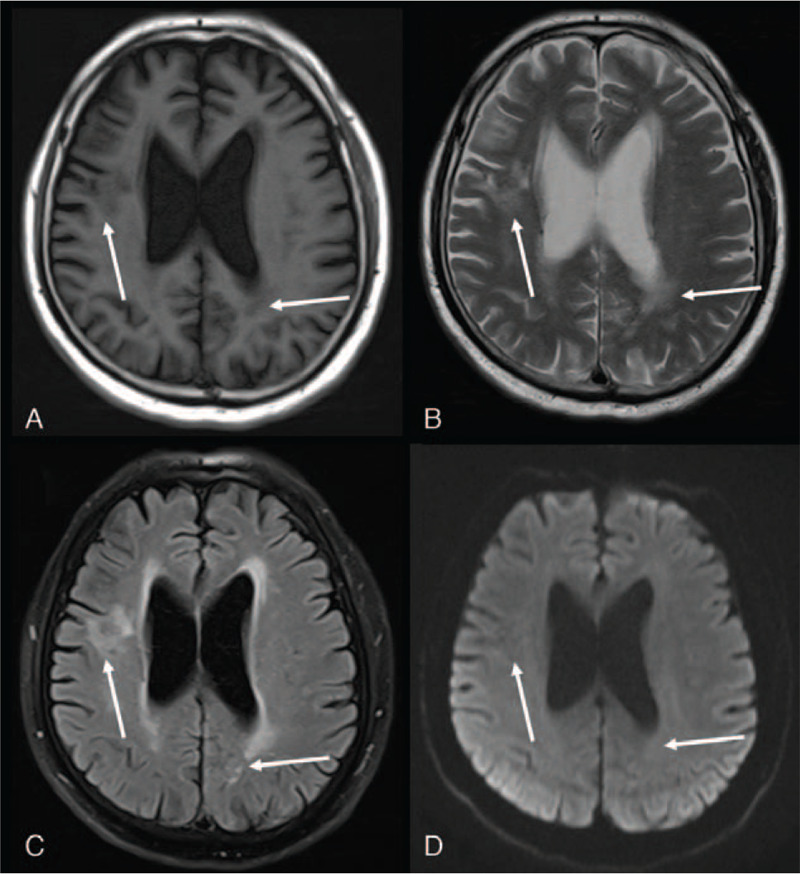
Reexamination of the brain MRI one and a half years later showed resolution of lesions with small softened foci and glial hyperplasia. A, Axial T1-weighted image. B, Axial T2-weighted image. C, Axial T2 Flair-weighted image. D, Diffusion-weighted image (b = 800). MRI = magnetic resonance imaging.

The patient recovered after a few days of intravenous anti-infective treatment, and was discharged eventually. Follow-up visits at the outpatient clinic showed no lasting neurological deficits. Reexamination of the brain MRI (Fig. 3) 1 and a half years later showed resolution of the lesions with small softened foci and glial hyperplasia.

## 3 Discussion

Contrast-induced encephalopathy is a very rare complication of intravascular contrast media. Most of the CIE cases are reported after arterial applications of contrast media such as cerebral or coronary angiographies. Nevertheless, only a few articles have reported CIE secondary to intravenous contrast.^[5–7]^

The exact mechanism behind neurotoxicity is still controversial. It has been postulated that a combination of chemotoxic effects related to contrast hyperosmolality and direct neurotoxicity of the contrast agent itself are responsible for its clinical presentation.^[8]^ The blood-brain barrier is impermeable to contrast agents under normal conditions. One of the theories regarding blood-brain barrier disruption is that hyperosmolar/ionic contrast agents can remove liquid from endothelial cells thus cause them to shrink and result in widened tight junctions.^[8,9]^ However, CIE has been reported less frequently with iso-osmolar non-ionic contrast agents.^[9,10]^ Some investigators suggested the mechanism of contrast-induced vessel wall irritation which leads to diffuse vasospasm and resultant hypoperfusion.^[11]^

The clinical presentations of CIE ranges from mild symptoms such as headache and vomiting to serious ones such as localized cortical and subcortical deficits (eg, hemiparesis, hemianopia, cortical blindness, aphasia, and parkinsonism) and global impairments (eg, seizure, and coma). Yet these symptoms always spontaneously resolve in hours to days.^[8,9,11]^ Transient cortical blindness is the most common manifestation of CIE, and altered mental status is a common precursor to neurological symptoms.^[12]^ Furthermore there have been a few cases where iodinated contrast injection led to a decline in consciousness.^[3]^

What is notable about our case is that the acute onset of dysarthria, unconsciousness and cardiorespiratory arrest which resulted after the administration of iso-osmolar contrast compound. Similarly, it has been previously reported that CIE can be secondary to contrast-enhanced CT examination with contrast agents of dosage 50 mL, 70 mL, and 120 mL.^[5–7]^ This reinforces a concept that a severe manifestation of CIE can occur due to any injection dosage of any type of iodinated contrast media.^[3]^ Nagamine Y et al reviewed the literature and pointed out that there is no clear connection between the incidence of CIE and the amount of contrast agent, while the amount of contrast agent used in the reported cases varied from 12 mL to 300 mL (mean, 125 mL).^[13]^ Some studies showed a correlation between contrast agent dosage and CIN. However, the exact maximal recommended dose is still unknown.^[11]^

Imaging examinations, involving CT and MRI, are important tools to distinguish CIE from other neurological emergencies such as cerebral hemorrhage, cerebral infarction and subarachnoid hemorrhage (SAH). Typical plain brain CT manifestations of CIE include diffuse cortical and subcortical enhancement, focal hyperdensity lesions, cerebral sulci hyperdensity, cerebral edema and subarachnoid space hyperdensity mimicking SAH.^[3,8,9,12]^ Hounsfield unit (HU) measurements may help differentiate SAH from CIE. The HU of blood is around 30 to 45 HU, while the HU of contrast agent usually is around 80 to 160 HU.^[9]^ However, some reports showed that asymptomatic contrast enhancement and cortex edema are common complications reported in 23% to 54% of CTs performed within 2 hours of uneventful embolization of cerebral aneurysms.^[14]^ Hence there is no uniform diagnostic criteria for CIE on CT. MRI findings of CIE include hyperintensity on T2 (spin–spin relaxation time), FLAIR (fluid-attenuated inversion recovery), and diffusion weighted images.^[8,12]^ However, CIE with absence of radiological findings have also been reported.^[12,15]^ In our case, the plain brain CT showed abnormal cortical contrast enhancement and cerebral sulci hyperdensity, which was rapidly resolved a day after, which is consistent with other reports.

The prognosis of CIE is generally favorable with rapid recovery over a few days. Even with severe manifestations like coma, patients can spontaneously recover from neurological symptoms.^[8]^ There were only a few rare cases reported with persistent deficits.^[14]^ The factors affecting the prognosis remain unknown.

Although there is no definitive prevention and treatment for this complication, adequate hydration with intra-arterial saline infusion during angiography and intravenous fluids are recommended. Intravenous steroids and mannitol for the control of cerebral edema may be helpful in some patients.^[10,16]^

In summary, our case illustrated a serious complication with cardiorespiratory arrest after intravenous contrast agent administration. After we ruled out other differential diagnoses like electrolytes imbalance, hypo/hyperglycemia, cardiogenic pathologies and neurological emergencies such as cerebral hemorrhage, cerebral infarction, a diagnosis of CIE was made. In addition, his recovery process was further complicated by brain abscesses as identified by MRI, although there is no evidence that brain abscess is related to the contrast agent. Fortunately, the patient soon recovered after a series of supportive treatments.

## 4 Conclusions

In conclusion, we reported a rare case of CIE following the administration of iso-osmolar contrast medium for chest CT examination. Our case suggested that CIE should be considered as 1 of the differential diagnoses for patient with acute neurologic episode after iodinate contrast administration. Neuroradiological imaging examinations are essential in ruling out other diseases such as acute cerebral infarction or intracranial hemorrhage.

## Author contributions

**Data curation**: Li-Ding Yao, Xiu-Liang Zhu.

**Writing – original draft**: Li-Ding Yao.

**Writing – review and editing**: Run-Lin Yang, Min-Ming Zhang.

## Abbreviations

Abbreviations: CIE = contrast-induced encephalopathy, CT = computed-tomography, HU = Hounsfield units, MRI = magnetic resonance imaging, SAH = subarachnoid haemorrhage.
